# Effect of 0.02% and 0.01% atropine on ocular biometrics: A two-year clinical trial

**DOI:** 10.3389/fped.2023.1095495

**Published:** 2023-01-17

**Authors:** Ming Wang, Can Cui, Shi-Ao Yu, Ling-ling Liang, Jing-Xue Ma, Ai-Cun Fu

**Affiliations:** ^1^ Department of Ophthalmology, The First Affiliated Hospital of Zhengzhou University, Zhengzhou, China; ^2^ Department of Ophthalmology, The Second Affiliated Hospital of Zhengzhou University, Zhengzhou, China; ^3^ Department of Ophthalmology, Shi Jiazhuang Aier Eye Hospital, Shi Jiazhuang, China

**Keywords:** myopia, children, efficacy, ocular biometrics, low-concentration atropine

## Abstract

**Background:**

Several studies have shown that various concentrations of low-concentration atropine can reduce myopia progression and control axial elongation safely and efficiently in children. The aim of this study was to evaluate the effects of 0.02% and 0.01% atropine on ocular biometrics.

**Methods:**

Cohort study. 138 and 142 children were randomized to use either 0.02% or 0.01% atropine eye drops, respectively. They wore single-vision (SV) spectacles, with one drop of atropine applied to both eyes nightly. Controls (*N* = 120) wore only SV spectacles. Ocular and corneal astigmatism were calculated using Thibos vector analysis and split into J0 and J45.

**Results:**

The changes in cycloplegic spherical equivalent refraction (SER) and axial length (AL) were −0.81 ± 0.52D, −0.94 ± 0.59D, and −1.33 ± 0.72D; and 0.62 ± 0.29 mm, 0.72 ± 0.31 mm, and 0.89 ± 0.35 mm in the 0.02% and 0.01% atropine and control groups, respectively (all *P* < 0.05). Both anterior chamber depth (ACD) and ocular astigmatism (including J0) increased, and lens power decreased in the three groups (all *P* < 0.05). However, there were no differences in the changes in ACD, ocular astigmatism, and lens power among the three groups (all *P* > 0.05). Intraocular pressure (IOP), corneal curvature, ocular astigmatism J45, and corneal astigmatism (including J0 and J45) remained stable over time in the three groups (all *P* > 0.05). The contributions to SER progression from the changes in AL, lens and corneal power of the three groups were similar (*P* > 0.05). The contribution of AL change alone to the change in SER was 56.3%, 63.4% and 78.2% in the above corresponding three groups.

**Conclusions:**

After 2 years, 0.02% and 0.01% atropine had no clinical effects on corneal and lens power, ocular and corneal astigmatism, ACD or IOP compared to the control group. 0.02% and 0.01% atropine helped to control myopia progression mainly by reducing AL elongation.

## Introduction

Myopia, also known as short-sightedness, continues to worsen over time because of changes in lifestyle and behavior, leading to high myopia. Prominently, high myopia, also called pathologic myopia, is relevant to excessive axial length (AL) growth, which can exponentially increase a person's risk of developing sight-threatening eye diseases such as macular hemorrhage, retinal detachment, glaucoma and cataracts (1–3). Therefore, effective treatment controlling myopia progression is critically important to preserve eye health and quality of life.

Several studies have shown that various concentrations of low-concentration atropine can reduce myopia progression and control axial elongation safely and efficiently in children (4–12). To date, five studies (5, 6, 11, 13, 14) on the efficiency of low-concentration atropine in managing myopia progression have been conducted over two years. However, there were different results regarding whether the effects of low-concentration atropine were similar in the second and first years; two 2-year studies (6, 11) found that the second-year efficacies of low-concentration atropine were similar to the first year, and another 2-year study (13) found that low-concentration atropine was more effective in the second year than in the first year.

Currently, only one 1-year study (15) found that low-concentration atropine had no effects on corneal and lens power, and antimyopic impact of low-concentration atropine acted mainly to reduce AL elongation. However, how ocular biometrics change and which ocular biometrics are associated with the antimyopic effects of low-concentration atropine for a longer time are unknown. In this prospective two-year cohort trial, the changes in ocular biometrics [including ocular and corneal astigmatism after vector analysis, corneal and lens power, intraocular pressure, and anterior chamber depth (ACD)] and their respective contributions to spherical equivalent refraction (SER) (i.e., spherical power plus 1/2 cylindrical power) progression were evaluated in children using 0.02% and 0.01% atropine compared with a control group.

## Material and methods

### Data source

The research approach has been published previously and is briefly described here (6, 7). Four hundred Chinese children with myopia (Han nationality, right eyes) who attended the First Affiliated Hospital of Zhengzhou University were enrolled on this cohort study between July 2016 and June 2018. The inclusion criteria were as follows: (1) 6–14 years of ages; (2) SER from −1.25 to 6.00 D; (3) astigmatism of <2.0 D; (4) anisometropia of <1.0 D; (5) monocular best-corrected visual acuity of 16/20 or better; (6) intraocular pressures (IOP) between 10 and 21 mmHg. There were no other eye diseases or past surgery. The exclusion criteria were previous use of atropine, pirenzepine, rigid gas-permeable, and orthokeratology lenses to reduce the progression of myopia and failure to adhere to the study's follow-up schedule.

Eligible children had the option of atropine or no atropine, and then the atropine groups were subsequently divided into either 0.01% or 0.02% in a double-blinded and randomized manner. This study conforms to the ethical norms and standards in the Declaration of Helsinki. The possible risks were thoroughly explained before the commencement of the treatment.

One clinician examined all subjects, who was unaware of the experimental groups of each participant. The children in the control group wore the full-correction single-vision (SV) spectacles with the least negative power, consistent with the best visual acuity of long-term wearing. Both experimental groups were prescribed SV spectacles according to the same protocol as the control group. The children in the experimental groups were given one drop of atropine eye drops in both eyes every night at bedtime.

After instilling one drop of compound tropicamide in both drops (0.5% tropicamide and 0.5% neo-synephrine) (Santen, Japan) 4 times at 10-minute intervals. Cycloplegic autorefraction was performed 10 min after the last administration of the eyedrop. Taking three autorefraction measurements (Topcon RM 8000A, CA, United States), the mean was obtained. The degree of myopia was expressed as the SER. Using Thibos vector analysis (16–18) calculated ocular and corneal astigmatism(minimum-maximum keratometry), which were split into its power vector components, J0 (with-the-rule astigmatism) and J45 (oblique).The ocular and corneal astigmatic components are denoted as OJ0, OJ45 and CJ0, CJ45, respectively. A non-contact partial coherence interferometer (IOLMaster-500, Carl Zeiss, Germany) was used for biometric measurements (AL, ACD, and corneal curvature) after cycloplegia. Five successive measurements were taken on each occasion, and their mean was used as the representative value. The machine IOLMaster-500 had corrected by the model eye every month. The signal-to-noise ratio for AL readings is greater than 2.0 according to the manufacturer's recommendations. IOP was measured using non-contact tonometry (TX-10, Canon, Japan). Using the Bennett-Rabbetts formula (19) calculated the lens power.


$$P_{L,\mathrm{BR}}=\frac{L\left(S_{\mathrm{CV}}+K\right)-1000n}{\left(L-\mathrm{ACD}-c_{\mathrm{BR}}\right)\left(\frac{\mathrm{ACD}+c_{\mathrm{BR}}}{1000n}\left(S_{\mathrm{CV}}+K\right)-1\right)}$$

Here: *P_L_*_,BR_-lens power using Bennett–Rabbetts method; *L*-axial length, *S*_cv_-spherical equivalent refraction at the corneal vertex [*S*_cv_ = *S*/(1 − 0.014*S*), *S*-spherical refraction at spectacle back vertex plane]; *K*-corneal power, *n* = 1.336 (the refractive index of aqueous and vitreous humors); ACD-anterior chamber depth; *c*_BR_ = 2.564 mm (distance between thin lens position and anterior lens surface).

### Statistical analysis

Only data from the right eye was used for analysis. Continuous baseline variables were expressed as the mean ± SD and evaluated using an analysis of variance. The chi-squared test was used to compare the number of male and female. A generalized additive mixed model was used to estimate the longitudinal trend with time (at baseline, 12, and 24 months) for dependent variables such as SER, corneal curvature, ocular astigmatism (OJ0 and OJ45), and corneal astigmatism (CJ0 and CJ45), and differences in the rate of change among the three groups. The change represents the slope for each treatment group of dependent variables over time, and the change difference represents the difference in the slope of dependent variables over time between the groups. Multivariate linear regression analysis was used to detect the relationship between the dependent variable (change in SER) and independent variables (sex, baseline age and changes in AL, lens power, corneal curvature, ACD, IOP). The adjusted *R*^2^ value was used to represent the proportion of the variance of the dependent variable predicted by the independent variable in the regression model. Statistical analysis was performed using the Empower software (www.empowerstats.com); X & Y Solutions, Boston, MA, United States) and SPSS 25.0 (IBM SPSS, Chicago, United States). Statistical significance was set at *P *< 0.05.

## Results

**Figure 1:**
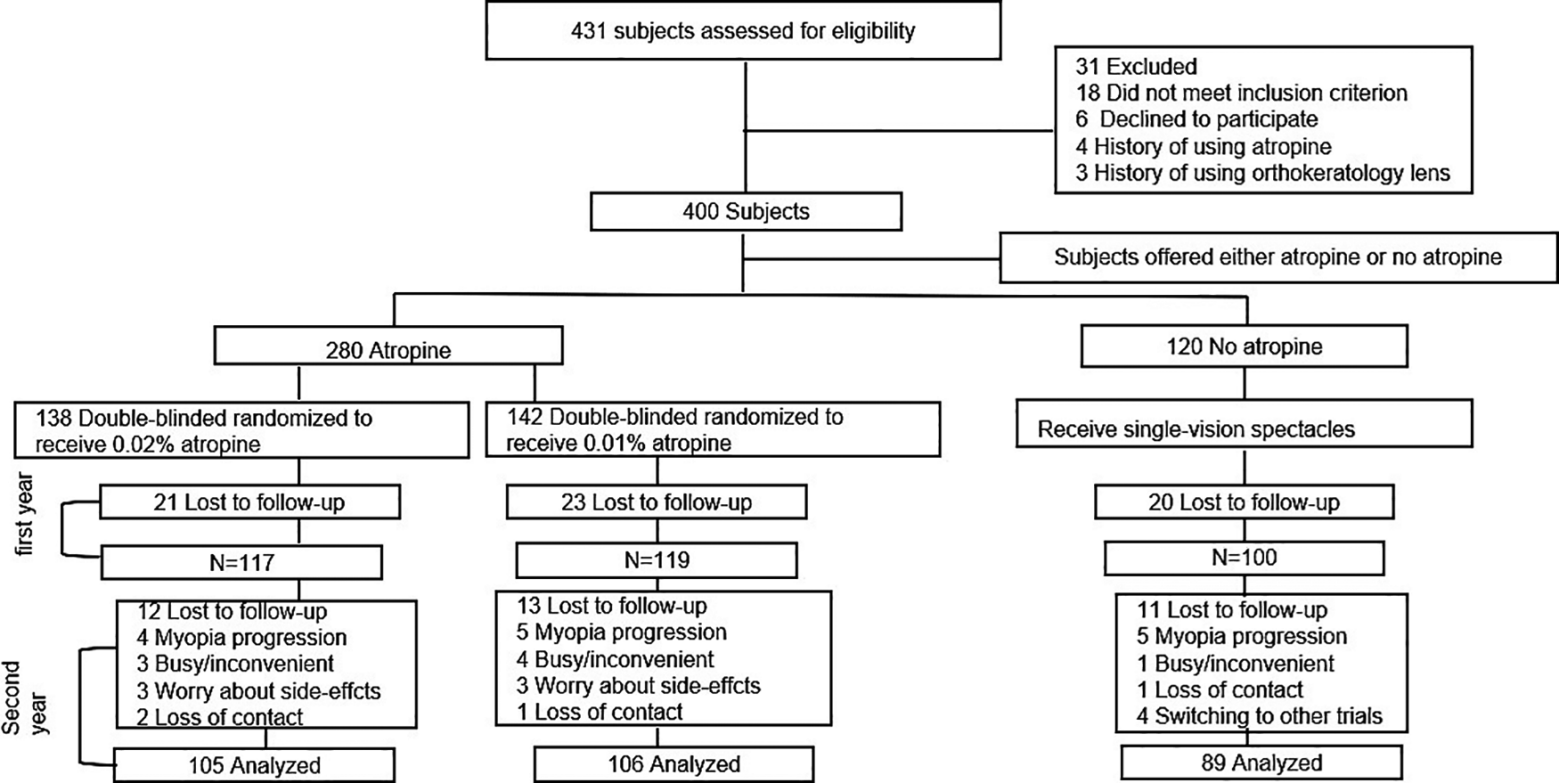
Subject recruitment and randomization flowchart.

In total, there were 138, 142, and 120 children in the 0.02% atropine, 0.01% atropine, and control groups, respectively (Figure 1). There were no differences in any of the baseline parameters between the three groups (Table 1).

**Table 1:** Baseline characteristics of study participants who completed 2 years vs. those who have not completed 2 years.

Variables	Completed 2 years (*N* = 300)				Not completed 2 years (*N* = 100)			
	0.02% atropine (*N* = 105)	0.01% atropine (*N* = 106)	control group (*N* = 89)	*P* Value	0.02% atropine (*N* = 33)	0.01% atropine (*N* = 36)	control group (*N* = 31)	*P* Value
Age (year)	9.6 ± 1.8	9.4 ± 1.7	9.3 ± 1.4	0.56	9.3 ± 2.1	9.2 ± 2.5	9.6 ± 2.3	0.66
Sex (male, *n* and %)	55 (52.4%)	55 (51.9%)	47 (52.8%)	0.99	18 (54.5%)	20 (55.6%)	15 (48.4%)	0.82
Spherical equivalent refractive errors (D)	−2.81 ± 1.47	−2.76 ± 1.56	−2.66 ± 1.39	0.62	−2.70 ± 1.79	−2.65 ± 1.88	−2.72 ± 1.75	0.58
Axial length (mm)	24.61 ± 0.69	24.60 ± 0.72	24.54 ± 0.69	0.80	24.58 ± 0.76	24.56 ± 0.79	24.56 ± 0.80	0.58
Anterior chamber depth (mm)	3.69 ± 0.20	3.70 ± 0.20	3.66 ± 0.21	0.92	3.62 ± 0.26	3.74 ± 0.27	3.69 ± 0.31	0.88
Intraocular pressure (mmHg)	15.9 ± 3.1	16.9 ± 2.8	17.0 ± 3.0	0.38	15.9 ± 3.1	16.9 ± 2.8	17.0 ± 3.0	0.42
Lens power (D)	22.75 ± 1.54	22.73 ± 1.41	22.78 ± 1.49	0.80	22.76 ± 1.52	22.75 ± 1.43	22.77 ± 1.51	0.80
Flattest *K* (D)	42.79 ± 1.50	42.81 ± 1.33	42.90 ± 1.09	0.76	42.81 ± 1.56	42.83 ± 1.44	42.94 ± 1.32	0.81
Steepest *K* (D)	43.98 ± 1.61	43.98 ± 1.45	44.02 ± 1.21	0.64	43.94 ± 1.65	43.99 ± 1.58	44.08 ± 1.46	0.40
**Ocular astigmatism (D)**								
Total	−0.43 ± 0.49	−0.42 ± 0.54	−0.34 ± 0.47	0.42	−0.41 ± 0.45	−0.41 ± 0.51	−0.36 ± 0.46	0.07
J0	0.16 ± 0.25	0.19 ± 0.27	0.17 ± 0.23	0.54	0.18 ± 0.25	0.17 ± 0.25	0.20 ± 0.24	0.07
J45	0.00 ± 0.15	−0.01 ± 0.09	0.00 ± 0.06	0.71	−0.01 ± 0.12	−0.02 ± 0.09	−0.01 ± 0.08	0.99
**Corneal astigmatism (D)**								
Total	−1.15 ± 0.53	−1.22 ± 0.61	−1.12 ± 0.40	0.32	−1.18 ± 0.55	−1.20 ± 0.60	−1.13 ± 0.40	0.55
J0	−0.53 ± 0.26	−0.58 ± 0.30	−0.54 ± 0.21	0.07	−0.60 ± 0.28	−0.62 ± 0.33	−0.59 ± 0.19	0.07
J45	0.04 ± 0.21	0.06 ± 0.18	0.02 ± 0.13	0.31	0.04 ± 0.20	0.05 ± 0.16	0.04 ± 0.15	0.49

### Changes in SER and AL over the two-year period

**Figure 2:**
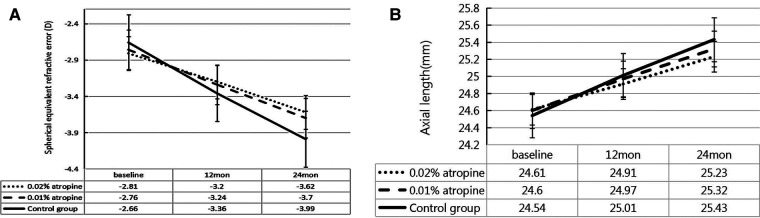
Measurement of spherical equivalent refractive error (**A**) and axial length (**B**) over time.

Over the two-year period, the changes in SER were −0.81 ± 0.52 D, −0.94 ± 0.59 D and −1.33 ± 0.72 D and changes in AL were 0.62 ± 0.29 mm, 0.72 ± 0.31 mm, and 0.89 ± 0.35 mm in the 0.02% and 0.01% atropine, and control groups, respectively. There were significant differences in the changes in SER and AL among the three groups (all *P *< 0.05) (Table 2 and Figure 2). In the 0.02% and 0.01% atropine and control groups, the changes in SER were −0.39 ± 0.35 D, −0.48 ± 0.45 D, and −0.70 ± 0.60 D, respectively, during the first year of treatment and −0.42 ± 0.32 D, −0.46 ± 0.45 D, and−0.63 ± 0.59 D, respectively, during the second year of treatment. The changes in AL were 0.30 ± 0.21 mm, 0.37 ± 0.22 mm, and 0.47 ± 0.35 mm during the first year of treatment and 0.32 ± 0.21 mm, 0.35 ± 0.22 mm, and 0.42 ± 0.34 mm during the second year in the three groups, respectively. The changes in SER during the first year in the three groups (all *P *> 0.05), and AL showed a tendency similar to that of SER every year.

**Table 2:** Change and change difference of ocular biometrics in three groups over 2-year^a^, mean (95% CI).

Variables	0.02% atropine		0.01% atropine		Control group		Change difference between-group			
	Baseline	24 months change	Baseline	24 months change	Baseline	24 months change	0.02% vs. 0.01% atropine	*P*-value	0.01% atropine vs. control group	*P*-value
Spherical equivalent refractive error (SER)	−2.81 (−2.90 to −2.72)	−0.12* (−0.19 to −0.05)	−2.76 (−2.81 to −2.71)	−0.17* (−0.25 to −0.09)	−2.66 (−2.70 to −2.62)	−0.24* (−0.28 to −0.17)	0.05 (0.01 to 0.07)	0.02	0.07 (0.04 to 0.10)	0.04
Axial length (AL)	24.61 (24.48 to 24.74)	0.10* (0.02 to 0.18)	24.60 (24.48 to 24.72)	0.15* (0.05 to 0.25)	24.54 (24.41 to 24.67)	0.26* (0.11 to 0.41)	0.05 (0.01 to 0.09)	0.04	0.11 (0.04 to 0.18)	0.002
Anterior chamber depth	3.69 (3.22 to 4.16)	0.08* (0.03 to 0.13)	3.70 (3.32 to 4.08)	0.06* (0.02 to 0.10)	3.66 (3.29 to 4.03)	0.06* (0.03 to 0.09)	0.01 (−0.01 to 0.02)	0.58	−0.04 (−0.09 to 0.02)	0.83
Intraocular pressure	15.9 (15.1 to 16.7)	−0.09 (−0.17 to 0.01)	16.9 (16.2 to 17.6)	−0.16 (−0.35 to 0.03)	17.0 (16.2 to 17.8)	−0.12 (−0.25 to 0.02)	−0.07 (−0.16 to 0.02)	0.12	0.04 (−0.01 to 0.08)	0.23
Lens power	22.75 (22.5 to 23.0)	−0.04* (−0.07 to −0.01)	22.73 (22.33 to 23.13)	−0.04* (−0.08 to −0.01)	22.78 (22.53 to 23.03)	−0.04* (−0.07 to −0.02)	0.005 (−0.003 to 0.012)	0.56	−0.002 (−0.006 to 0.002)	0.66
Flattest K	42.79 (42.44 to 43.14)	−0.05 (−0.10 to 0.01)	42.81 (42.46 to 43.16)	−0.06 (−0.14 to 0.02)	42.90 (42.51 to 43.29)	−0.10 (−0.26 to 0.04)	−0.01 (−0.04 to 0.02)	0.45	−0.04 (−0.08 to 0.01)	0.19
Steepest K	43.98 (43.58 to 44.38)	0.08 (−0.03 to 0.19)	43.98 (43.52 to 44.44)	0.09 (−0.03 to 0.21)	44.02 (43.72 to 44.32)	0.06 (−0.02 to 0.14)	0.01 (−0.03 to 0.05)	0.51	−0.02 (−0.05 to 0.01)	0.33
Total ocular astigmatism	0.16 (0.10 to 0.22)	−0.19* (−0.26 to −0.12)	0.19 (0.12 to 0.26)	−0.23* (−0.35 to −0.11)	0.17 (0.11 to 0.23)	−0.20* (−0.30 to −0.10)	−0.03 (−0.06 to 0.01)	0.26	0.03 (−0.02 to 0.08)	0.58
Ocular astigmatism (J0)	0.16 (0.13 to 0.19)	0.09* (0.06 to 0.12)	0.19 (0.14 to 0.24)	0.11* (0.05 to 0.17)	0.17 (0.08 to 0.26)	0.08* (0.02 to 0.14)	0.02 (−0.01 to 0.05)	0.35	−0.03 (−0.06 to 0.01)	0.47
Ocular astigmatism (J45)	0.00 (−0.01 to 0.00)	−0.01 (−0.03 to 0.01)	−0.01 (−0.04 to 0.02)	−0.01 (−0.03 to 0.01)	0.00 (−0.03 to 0.03)	−0.01 (−0.03 to 0.01)	−0.01 (−0.04 to 0.02)	0.10	0.01 (−0.01 to 0.03)	0.24
Total corneal astigmatism	−1.15 (−1.45 to −0.85)	−0.10 (−0.25 to 0.05)	−1.22 (−1.32 to −1.12)	−0.14 (−0.31 to 0.03)	−1.12 (−0.82 to −1.42)	−0.13 (−0.33 to 0.07)	−0.04 (−0.08 to 0.01)	0.37	0.01 (−0.02 to 0.04)	0.95
Corneal astigmatism (J0)	−0.53 (−0.45 to −0.61)	0.02 (−0.01 to 0.04)	−0.58 (−0.41 to −0.75)	−0.04 (−0.10 to 0.02)	−0.54 (−0.24 to −0.84)	−0.03 (−0.07 to 0.01)	−0.06 (−0.13 to 0.01)	0.28	0.02 (−0.01 to 0.05)	0.75
Corneal astigmatism (J45)	0.04 (0.01 to 0.07)	0.003 (−0.001 to 0.007)	0.06 (0.02 to 0.10)	−0.005 (–0.01 to 0.01)	0.02 (0 to 0.04)	0.005 (−0.001 to 0.010)	0.01 (−0.01 to 0.03)	0.78	−0.02 (−0.05 to 0.01)	0.48

Note: CI, confidence interval. Change: represents the slope of SER, AL, and other ocular biometrics over time for three groups. Change difference: represents the difference in SER, AL, and other ocular biometrics over time between each two groups.

^a^ A generalized additive mixed model was used to estimate the longitudinal trend from baseline to 24 months.

* Represents: changes were significantly different.

### Changes in other ocular biometric parameters over the two-year period

**Figure 3:**
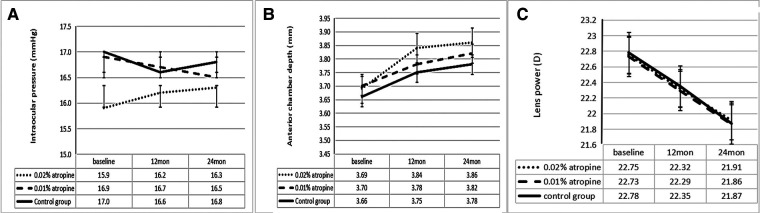
Measurement of intraocular pressure (**A**) and anterior chamber depth (**B**) and lens power (**C**) over time.

**Figure 4:**
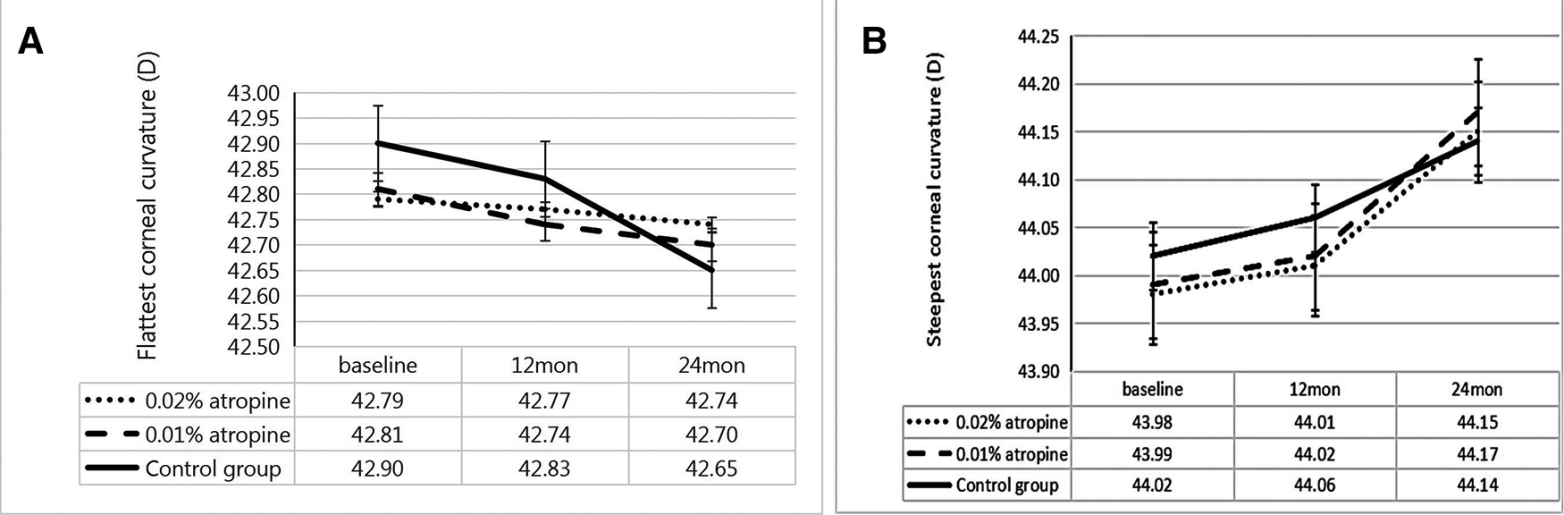
Measurement of flattest corneal curvature (**A**) and steepest corneal curvature (**B**) over time.

**Figure 5:**
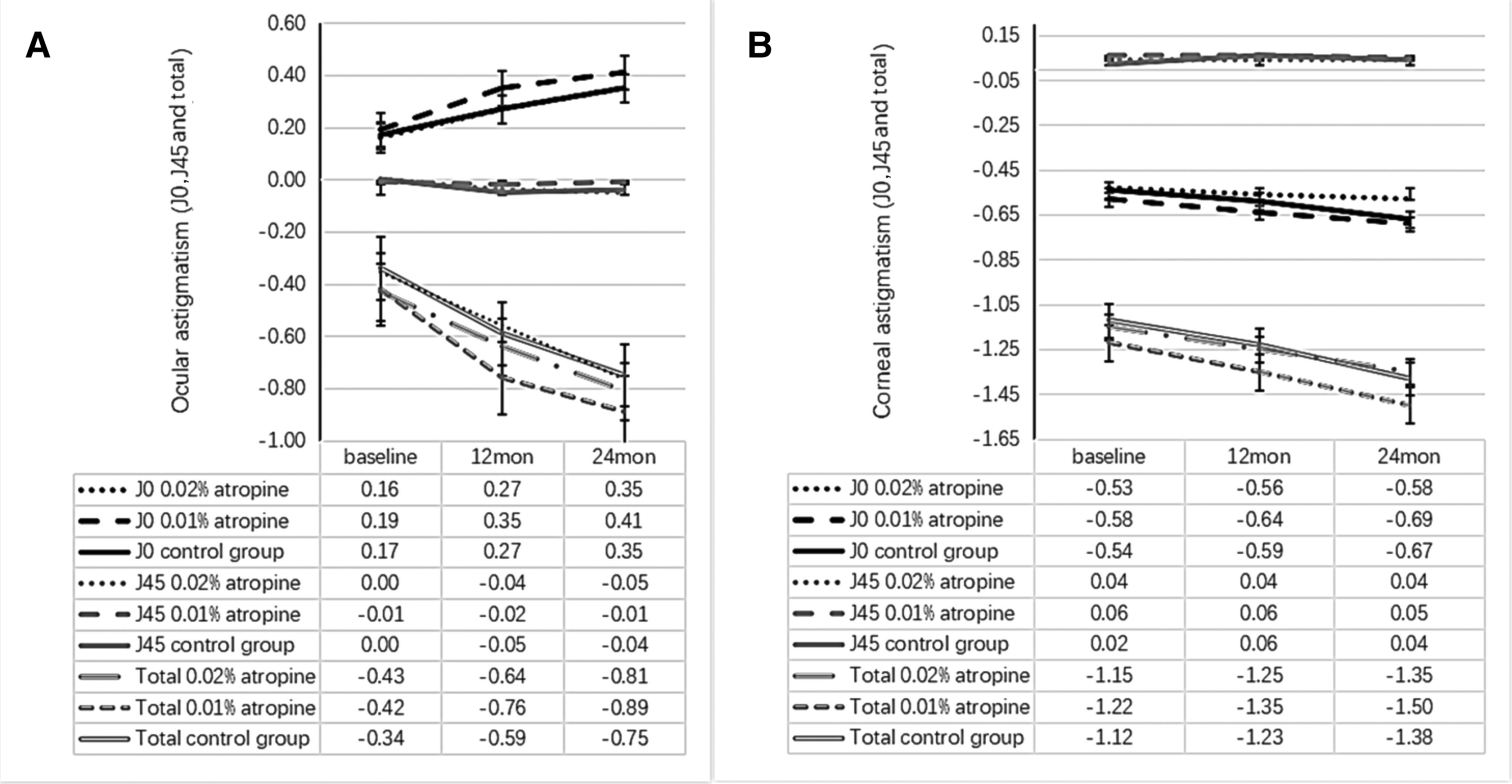
Measurement of ocular astigmatism (J0, J45 and total) (**A**) and corneal astigmatism (J0, J45 and total) (**B**) over time.

The changes in ACD, IOP, corneal curvature, astigmatism, and lens power in the 0.02% and 0.01% atropine and control groups are summarized in Table 2 and Figures 3–5. The IOP, flattest, and steepest corneal curvature remained stable over time before and after treatment, and there were no significant differences in the changes in IOP, flattest, and steepest corneal curvatures (Figures 3, 4) among the three groups (all *P *> 0.05). The ACD increased over time in all three groups, but the degree of change was similar (Figure 3). Lens power decreased over time in each group, but the differences were identical (Figure 3).

The ocular astigmatism changes were −0.38 ± 0.29 D, −0.47 ± 0.38 D, −0.41 ± 0.35 D and ocular astigmatism J0 changes were 0.19 ± 0.28 D, 0.22 ± 0.36 D, 0.18 ± 0.31 D in the 0.02%, 0.01% atropine and control groups, respectively. There was a small but significant increase in ocular astigmatism (including J0) (all *P *< 0.05) but no change in J45 (all *P *> 0.05; Figure 5) in the three groups. However, there were no significant differences in the changes in ocular astigmatism (including J0) among the three groups. The corresponding changes in corneal astigmatism were −0.20 ± 0.34 D, −0.28 ± 0.35 D, and −0.26 ± 0.26 D in the three groups. There was no significant increase or difference in the change in corneal astigmatism (including J0 and J45) among the three groups (all *P *> 0.05).

The contributions of ocular biometric changes (sex, baseline age and changes in AL, lens power, corneal curvature, ACD, IOP) to SER progression were summarized in Table 3. Myopia progression was mainly caused by AL elongation, which contributed 56.3%,63.4% and 78.2% of the myopia progression in the 0.02%, 0.01% atropine and control groups, followed by corneal and lens power. The relationship between the changes in AL (independent variable) and SER (dependent variable) was −1.71, −1.65 and −2.09 in the above corresponding three groups (all *P *< 0.001). Considering the ocular biometric changes (changes in AL, lens power, corneal curvature, ACD, IOP), sex, and age, the multiple linear regression model explained 80.6%, 77.1%, 84.7% of the SER change in the three groups (Model 4).

**Table 3:** Multivariate linear regression analyses of change in spherical equivalent refractive error and ocular biometrics.

Variables	0.02% atropine			0.01% atropine			Control group		
	*β*	Standard error	*P* value	*β*	Standard error	*P* value	*β*	Standard error	*P* value
**M1**									
ΔAL (mm)	−1.71	0.24	<0.001	−1.65	0.20	<0.001	−2.09	0.11	<0.001
Adjusted *R*^2^ (%)	56.3			63.4			78.2		
**M2**									
ΔAL (mm)	−2.23	0.21	<0.001	−1.80	0.19	<0.001	−2.20	0.10	<0.001
ΔLens power (D)	−0.30	0.06	0.001	−0.16	0.06	0.007	−0.29	0.06	<0.001
Adjusted *R*^2^ (%)	74.7			69.2			82.9		
**M3**									
ΔAL (mm)	−2.41	0.20	<0.001	−1.84	0.18	<0.001	−2.40	0.12	<0.001
ΔLens power (D)	−0.39	0.06	<0.001	−0.24	0.07	0.001	−0.36	0.06	<0.001
ΔCorneal power (D)	−0.60	0.22	0.009	−0.47	0.23	<0.001	−0.68	0.23	0.003
Adjusted *R*^2^ (%)	78.5			71.6			84.2		
**M4**									
ΔAL (mm)	−2.51	0.21	<0.001	−2.04	0.17	<0.001	−2.41	0.12	<0.001
ΔLens power (D)	−0.38	0.06	<0.001	−0.31	0.07	<0.001	−0.36	0.06	<0.001
ΔCorneal power (D)	−0.78	0.23	0.002	−0.51	0.16	0.002	−0.70	0.23	0.003
ΔACD (mm)	0.33	0.13	0.60	0.30	0.14	0.06	0.37	0.24	0.12
ΔIOP (mmHg)	0.03	0.01	0.02	−0.02	0.18	0.21	−0.002	0.004	0.71
Sex	0.16	0.07	0.81	−0.05	0.06	0.45	−0.005	0.02	0.85
Age	−0.03	0.02	0.25	−0.04	0.02	0.09	−0.02	0.007	0.03
Adjusted *R*^2^ (%)	80.6			77.1			84.7		

Note: Δ, change over 2 years; AL, axial length; ACD, anterior chamber depth; IOP, intraocular pressure.

## Discussion

This two-year cohort clinical trial showed that 0.02% atropine had a better effect on controlling myopia progression and axial elongation than 0.01% atropine. Additionally, no significant differences in the changes in corneal and lens power, ocular and corneal astigmatism, ACD, and IOP between the 0.02% atropine, 0.01% atropine, and control groups. Contributions to SER progression from AL, corneal and lens power in the three groups were similar, axial elongation contributed to most of the SER progression, and 0.02% and 0.01% atropine helped to control myopia progression mainly by reducing axial elongation.

Several studies (5, 11, 20, 21), have found that the efficacy of low-dose atropine for myopia control is concentration-dependent. The higher the concentration of low-dose atropine, the better the myopia progression control, and the less the axial elongation. Also, low-dose atropine synchronously controlled SER progression and AL elongation, as described in Hong Kong (11), Korea (21), and mainland China studies (6, 7). Although 0.02% and 0.01% atropine controlled the myopia progression and AL elongation synchronously, the control rate of AL elongation was less than that of myopia progression. This was consistent with other studies that explored the significantly higher control rate of SER progression than AL elongation in myopic children using low-dose atropine and whose baseline profiles were similar to those of the current study (7, 11, 15, 22, 23). There are two potential explanations for this finding. First, changes in AL were not the only factors causing changes in SER. LAMP study (15) found that AL alone contributed to an SER variance ranging from 72% to 81%. The remaining SER variance was accounted for by lens and corneal factors in myopic children using low-dose atropine. In the current study, we found no significant effect on corneal and lens power of 0.02% and 0.01% atropine compared with the control group and similar contributions to SER progression from AL, corneal and lens power between two atropine and control groups. The contribution of change in AL alone to the change in SER was 56.3% and 63.4% in 0.02% and 0.01% atropine groups, axial elongation contributed to most of the SER progression, and the remaining change in SER was accounted for by corneal and lens power, and other factors that have not yet been discovered. Second, children's AL elongation includes small average age-related growth and SER progression-associated growth (24, 25). This may partly explain why the effect of low atropine on total axial elongation is less than that of SER progression. Overall, the myopia control effects of 0.02% and 0.01% atropine in myopia children were mainly achieved by inhibiting axial elongation, and lens power, corneal curvature, and age were rarely involved.

Furthermore, 0.02% and 0.01% atropine did not affect corneal and ocular astigmatism (after vector decomposition) and ACD compared to with the control group in the current two-year study. LAMP study (15) also found that 0.05%, 0.025%, and 0.01% atropine had no significant effects on corneal astigmatism in the first year of treatment. However, the changes in ocular and corneal astigmatism are not vectorized.) Studies (26, 27) have found that the change in astigmatism was presumably because of the flattening and motorial of the lens during cycloplegia, which results from the axis tilt around the horizontal axis. When the atropine concentration was below 0.05%, it may have been too low to change the lens thickness and power and not increase astigmatism further. In addition, 0.02% and 0.01% atropine did not affect IOP in the current study, although pupillary dilation caused by low-dose atropine is a predisposing factor for high IOP. Other reports have also found that using different concentrations of atropine (e.g., 1%, 0.5%, 0.25%, 0.125%, and 0.1%) has no effect on IOP in myopic children (28–30). Overall, considering the ocular biometrics such as corneal curvature, lens power, corneal and ocular astigmatism, ACD, and IOP, 0.02% and 0.01% atropine all appear to be safe for children with myopia.

The strength of this study was to include a control group. The human ethics committee advised that subjects were to be offered either atropine or no atropine, and double-blinded randomization is carried out only for the two active arms of the study. The control and test groups had similar demographic and clinical parameters. The subjects were recruited using identical inclusion criteria contemporaneously and from the same population. A limitation of the current study is that a changing trend in ocular biological parameters after atropine withdrawal was not observed. This topic is currently under further investigation.

## Conclusion

Use of 0.02% and 0.01% atropine had no clinically significant effect on corneal curvature, corneal and ocular astigmatism, lens power, ACD, or IOP. 0.02% and 0.01% atropine mainly help control myopia by reducing axial elongation over the two-year follow-up period.

## Data Availability

The raw data supporting the conclusions of this article will be made available by the authors, without undue reservation.
